# Short-term impacts of embryonic thermal manipulation in mule duck, a kinetic study: new tools for metabolic programming

**DOI:** 10.1186/s12864-025-12192-7

**Published:** 2025-11-07

**Authors:** C. Andrieux, S. Biasutti, LL. Zwick, M. Marchand, S. Panserat, M. Houssier

**Affiliations:** 1 Université de Pau et des Pays de L’Adour, INRAE, NUMEA, Mont de Marsan, 40000 France; 2 Université de Pau et des Pays de L’Adour, IUT Génie Biologique, Mont de Marsan, 40000 France

**Keywords:** Mule duck, Thermal manipulation, Programming, Metabolism

## Abstract

**Background:**

Temperature changes during embryogenesis, through a process called embryonic thermal programming, can modify the long-term thermotolerance of broilers or the hepatic metabolism of mule ducks. This study focused on the very short-term impacts on mule duck livers of such a programming which consist of increasing the incubation temperature by + 1°C, 16h/24h, from the 13th to 27th day of embryonic development. Using fluidigm technology, we analysed the impact of this temperature change on the relative expression of 81 genes involved in various metabolic pathways closely or remotely related to the fattening of the liver of mule ducks.

**Results:**

Expression changes were first assessed during temperature increase (from 30 min to 7h, then 3 days, 7 days and 11 days after the start of modification), and at hatching (3 days after the end of temperature modification) in comparison with a group of animals not exposed to this programming.

First, zootechnical measurements confirmed that the moderate discontinuous increase in embryonic temperature led to a drop in internal temperature and hatch weight, but did not reduce hatchability.

Secondly, gene expression in all tested metabolic pathways was affected throughout the study, except for cell proliferation and epigenetic marks, which were only modulated during the thermal stimulus. The most strongly and durably modulated pathways, with significant changes in the expression of several genes at multiple sampling points, were lipid metabolism (*DGAT2, CEPT1, GPAT1, ACOX1*), cellular stress (*HSPA5-HSP70*), and thyroid hormone regulation (*NCOR*). Some genes such as *SCD1* (lipid synthesis), *DIO3* (thyroid hormone regulation), *HSPA2*-*HSP70, HSBP1, HSP90AA1* and *IL18* (cellular stress) also showed modulation even after the thermal stimulus ended. Interestingly, the expression of genes involved in epigenetic and cell proliferation was only slightly affected by the temperature increase, except for *ELP3* (epigenetic marks) which was significantly modulated at two points.

**Conclusion:**

This study is the first to show the short-term impact of increased egg incubation temperature on gene expression in mule duck liver, from the start of the stimulus to hatching. These results could provide a valuable starting point for understanding the mechanisms of embryonic thermal programming that modulate hepatic metabolism in mule ducks.

**Supplementary Information:**

The online version contains supplementary material available at 10.1186/s12864-025-12192-7.

## Introduction

Early environmental stimuli are capable of modifying phenotype, in the short or long term, in animal life [1]. This phenomenon, known as programming [2], has been used in animal production and more specifically in the poultry industry to improve breeding performance [3]. Various stimuli can be applied to program a phenotype such as different colored light during incubation [4–6], variable availability of specific nutrients in the egg [7, 8], or different egg incubation temperature [9, 10]. These early exposures can influence various physiological pathways, such as muscle growth [9–11], global health [12, 13] or the ability to withstand heat stress [14, 15]. In mule duck, it was recently shown that an increase in temperature, from 1 to 1.7 °C during embryogenesis, resulted in higher *foie gras* production after a 2-week-forced-fed [16, 17].

These phenotypic changes are generally associated with molecular changes such as protein expressions in broiler muscles [18], DNA methylations in broiler brain [19] or transcriptional modulations in chicken heart, brain or spleen [19–21] or in duck liver [16, 22]. The transcriptional effects of embryonic thermal programming are often measured at two stages of the process: firstly, just after the temperature change, and secondly, after the programmed phenotype has been revealed, i.e. often much later in the animal's life. For the first stage, the genes studied are mainly involved in stress perception, belonging in particular to the family of heat shock proteins [23], whereas for the second stage, measurements often focus on the expression of genes involved in revealing the programmed phenotype [17, 22, 24]. These measurements reflect the transcriptional responses at a particular, arbitrarily chosen time point following stimuli.

The objective of this study was to evaluate how embryonic thermal programming affects the expression of genes involved in diverse cellular pathways in the liver of mule ducks, beginning 30 min after the initial thermal stimulus, over several days during the programming phase, and 3 days following its end. To this end, mule duck eggs were subjected to a discontinuous (16h/24h) increase in temperature of 1 °C during the 13th to the 27th embryonic day (E13-E27) in parallel with a control group with no temperature change, before being transferred to the same hatchery. Mortality at different stages, final hatchability, sex ratio, internal temperature, and body weight were measured at hatching (E30). Gene expression was assessed at multiple time points: from 30 min to 7 h after the onset of the temperature increase at E13, then at 3, 7, and 11 days after the start of the temperature increase (E16, E20, and E24), corresponding to the programming period. A final gene expression measurement was taken 3 days after the end of the temperature increase, at hatching (E30).

## Materials and methods

### Animals

**Fig. 1:**
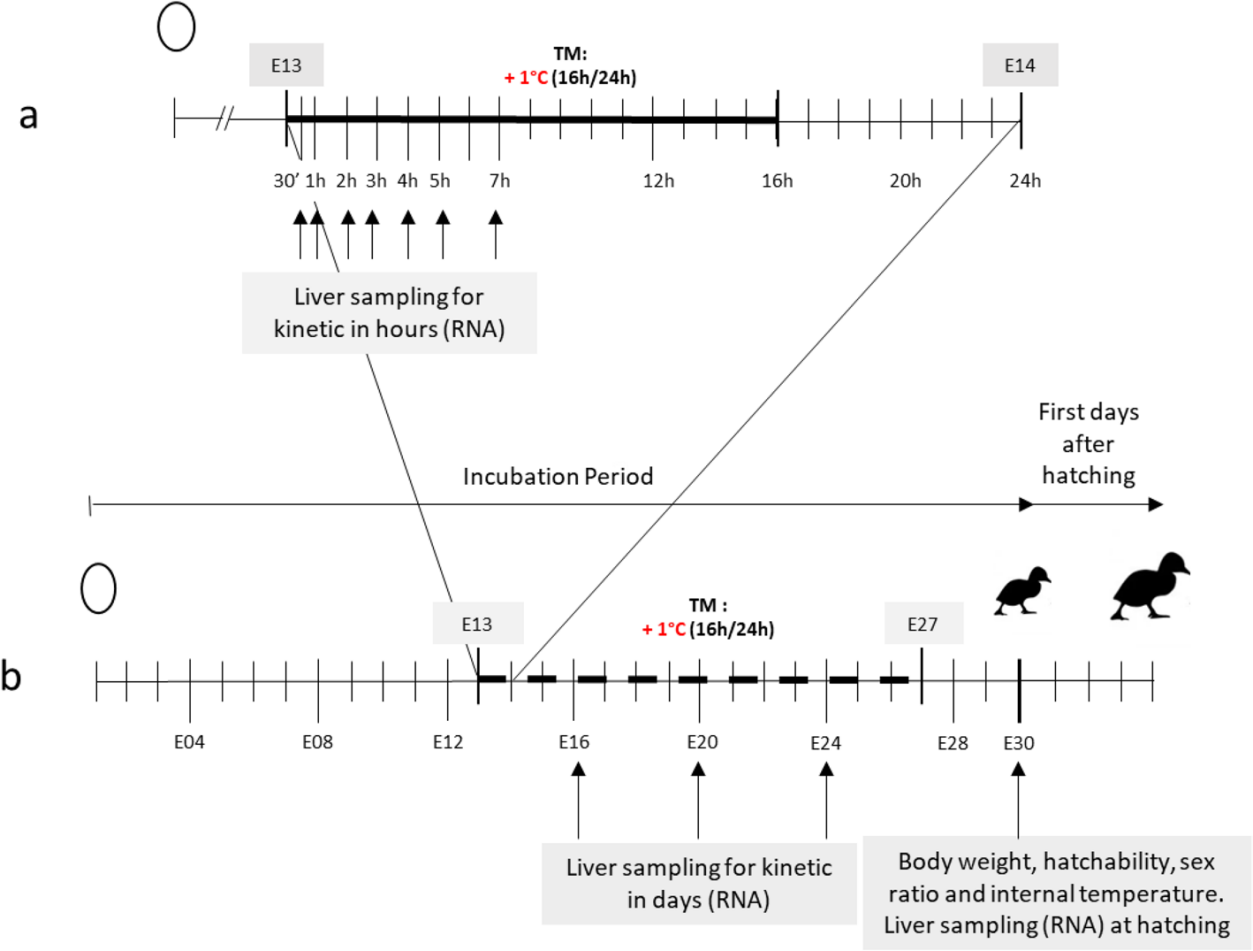
Experimental design. For both trials (**a** and **b**), the incubation temperature was maintained at 37.6°C throughout the incubation period for the control group. In the TM (thermal manipulated) group, a temperature increase of 1 °C, 16h/24 was applied from embryonic day 13 to 27 (E13-E27), illustrated by a bold dotted line. In the first trial (**a**), liver were sampled 30 min, 1 h, 2 h, 3 h, 4 h, 5 h and 7 h after the start of the thermal stimulus. In the second trial (**b**), liver samples were taken 3 days, 7 days and 11 days after the start of the temperature increase, corresponding to embryonic days 16, 20 and 24 (E16, E20 and E24). A final sample was taken at hatching (E30), 17 days after the start of the stimulus in both groups. At hatching, body weight, hatchability, sex ratio and internal temperature were also recorded

The experiment was divided into two individual trials (Fig. 1). For both trials, eggs from the two groups were collected on the same day and stored at room temperature for 3 days before incubation (genotype H85, provided by Grimaud Frères Selection company, Roussay, France). The eggs were randomly divided in two (second trial) or four (first trial) identical incubators for two incubation treatments (SOLOGNE model of LA NATIONALE reconditioned model with a capacity of 2,430 eggs). All incubators were maintained at a constant temperature of 37.6 °C with an average relative humidity of 63.2%, from the embryonic day 0 (E0) to embryonic day 13th (E13) (24h/24). Incubator temperature, relative humidity (RH) and ventilation were automatically regulated. Moreover, the temperature and the RH were confirmed by records every 15 min by two independent sensors (LoRa® SPY U) in each incubator. All eggs in both groups were subjected to automatic 90° rotation every three hours and were manually sprayed for daily cooling from embryonic day 8 until the end of incubation, by opening the incubator doors for a few minutes at 2 pm. This cooling was applied just before the daily temperature increase, with the aim of facilitating the concomitant rise in relative humidity for TM group.

For the first trial (Fig. 1a), at embryonic day 13 (E13), the temperature in two of the four incubators was increased by 1 °C from 8:00 a.m. with an average relative humidity (RH) of 60.9% (Thermal Manipulated or TM group, 250 eggs) while the control incubators were maintained at 37.6 °C and 60.1% RH (control group, 250 eggs). The eggs were then collected from the first two incubators (1 control and 1 TM incubator) at 30 min, 2 h, 4 h, and 7 h after the temperature increase, and from the other two incubators of each group at 1 h, 3 h, and 5 h, in order to avoid significant temperature fluctuations caused by opening the doors during sampling. The embryos were euthanized by decapitation, in accordance with ethical principles [25], to allow for the collection of liver samples. To ensure consistent handling conditions, the 15 eggs from each group were temporarily transferred to a mobile incubator maintained at 37.6 °C, and dissections were performed alternately: one control egg/one TM egg.

For the second trial (Fig. 1b), from E13 to E27 the temperature in one of the two incubators was increased by 1 °C each day from 4:00 p.m. to 8:00 a.m. with an average RH of 63.6% (TM group, 750 eggs), while the other incubator was maintained at 37.6 °C with 63.2% RH (control group, 750 eggs) throughout the entire incubation period. Liver samples were then taken at E16, E20 and E24 during the programming period at 5 p.m, 1 h after the start of daily temperature rise (when the incubation temperature was reached and stable). At day 28 (E28), a candling was performed to assess mid-embryonic mortality (mortality between embryonic day 7 to 28), and all viable eggs were moved into the same hatchery (BRETAGNE model of LA NATIONALE, reconditioned model with a capacity of 2,520 eggs) maintained at 37.3°C and 80% of RH for three days. From E28 to E30, newly hatched ducklings were recorded once per day. Unhatched ducklings at E30 were counted to determine late mortality, rate of fertilized eggs (not shown, (total number of eggs put in incubation – number of eggs unfertilized identified during candling)/total number of eggs put in incubation) and hatchability (calculated as the ratio of the total number of hatched ducklings to the number of fertilized eggs set in the hatcher, multiplied by 100) while final liver samples were taken from ducklings born at E30 in both groups, 3 days after the end of the programming period.

### Sampling and measurements

In the first trial whole livers (8 per condition) were collected 30 min, 1 h, 2 h, 3 h, 4 h, 5 h and 7 h after the first day of temperature increase (E13). In the second trial, whole livers (8 per condition) were collected 1 h after the daily temperature increase (5 p.m.), 3, 7 and 11 days after the temperature increase (E16, E20 and E24) and three days after the end of the temperature increase, at hatching (E30). Similar samples were taken at the same times for the control group, not exposed to the temperature increase. The livers were immediately immersed in liquid nitrogen before being stored at −80 °C.

The sex of all hatched ducklings (433 in the control group and 475 in the TM group), the body weight of dry male ducklings (237 in the control group and 255 in the TM group), and the cloacal temperature of approximately 100 male ducklings in each group were recorded at E30, by using digital express thermometers (Digital Express Thermometer from Gilbert).

### RNA extraction, reverse transcription, fluidigm and relative expression measurement

The TRIzol method (Invitrogen/Life technologies) was used to extract the total RNA from 50 mg of frozen liver tissue. Total RNA samples were normalized at 100 ng/µL after concentration analysis by spectrophotometry (optical density at 260 nm) using a Biotek EPOCH 2 microplate reader (Take 3 Plate). RNA reverse transcription (RT) was performed with 1µg of RNA mixed with Super Script (Life Technologies SAS) and RNAseOUT (Life Technologies SAS). The RT mix was completed by an exogenous RNA (Luciferase, Promega, 1 pg) to have an exogenous control. The RT was done in the CFX384 (BioRad, USA) according to the following program: 25°C/5 min, 55°C/60 min, 70°C/15 min.

Gene expression levels were determined by high throughput real-time quantitative PCR from Fluidigm (Gentyane platform, Clermont-Ferrand, France). Two 96 well plates were prepared, one with samples (cDNA) at 5 ng/µL and the second with gene primers at 20 µM, listed in SupplementTable 1. Primer validations were carried out in two steps: their efficiency ranging from 1.85 to 2 was calculated from cascade dilution of a cDNA pool and their specificity was confirmed by amplicon sequencing. The sample amplifications were realized in two steps by using the Fluidigm method. A first pre-amplification with Preamp Master Mix (Fluidigm) was done following program: 95 °C/10 min and 14 cycles of 95 °C/15 s and 60 °C/4 min. Pre-amplified samples were treated by exonuclease (NEB) before a low EDTA buffer (10mM Tris–HCL, 0.1 mM EDTA) dilution and before moving samples to a 96 × 96 chip for Fluidigm Gene Expression Array. The reaction was made using 20 × EvaGreen (Interchim) dye following the program:70 °C/2400 s, 60 °C/30 s, 30 cycles of 96 °C/5 s and 60 °C/20 s. Real time quantitative PCR results were analyzed using the Fluidigm real-time PCR analysis software v.4.1.3.

A initial list of genes of interest was compiled from previous studies conducted on different species concerning metabolic pathways of interest such as stress, inflammation or energy metabolism. 95 genes were selected for Fluidigm analysis and ultimately only those validated for specificity and efficiency were retained for gene expression analysis. Initially, 8 replicates per condition were deposited to calculate a relative expression mean, and only genes for which at least 6 replicates were finally detected were retained for statistical analysis. A total of 81 genes were kept for the hour and the daily kinetics.

The relative expression was normalized with four reference genes (*ActB*, *Luciferase*, *STAB1* and *USP9X* for the first trial, and *HPRT1*, *Luciferase*, *STAB1* and *UPS9X* for the second trial), selected according to the function GeNorm from the package CtrlGene of R (v.3.6.2). Moreover, the relative expression was calculated according to the expression 2^−∆∆Ct^ [26] with ∆Ct sample = Ct target gene – Ct reference genes (mean of 4 previous genes) and ∆∆Ct = ∆Ct sample—∆Ct reference. The reference that determined the ∆Ct reference value corresponded to the average of Ct across all groups.

### Statistical analysis

Statistical analyses were carried out using R & Rstudio version 3.6.2. Hatchability, sex ratio and mortalities were compared by using a Chi-squared test. Student (parametric) or Wilcoxon (non parametric) tests were done depending on normality and homoscedasticity. A student test was applied for body weights at hatch and a Wilcoxon test was done for internal temperatures at hatch. Tests have been adapted to compare the relative expression between groups for the two trials (*n* = 6 minimum) for each timing (2 ways analysis was excluded due to the flattening of the TM effects).

The data are presented as the average ± standard error of deviation (SD). In every case, differences between the groups were considered statistically significant when *p* < 0.05.

## Results

### Short-term transcriptional impacts of thermal manipulation in the liver

**Fig. 2:**
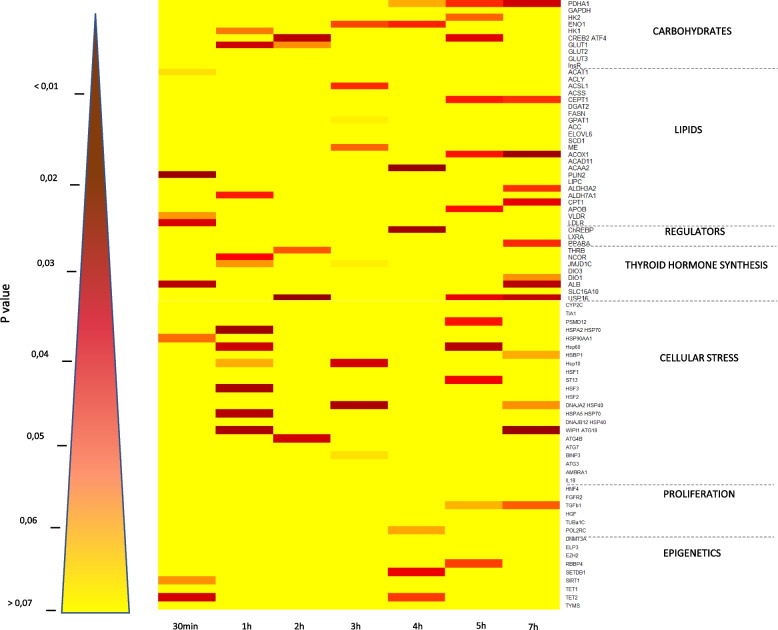
Hourly kinetics of hepatic gene expression at E13. The heat map represents an overview of the 81 genes studied, presented by cellular pathway for the 7 sampling points at E13, the first day of temperature increase (30min, 1 h, 2 h, 3 h, 4 h, 5 h and 7 h after the modification). Student or Wilcoxon tests were applied, depending on the conditions to be met, to compare the control and TM groups for the same sampling point. The absence of a significant difference is shown in orange/yellow, while red/brown indicates a significant difference between the two groups according to the color key

The first trial enabled the analysis of the very short-term transcriptional impact of an increase in incubation temperature, based on an hourly kinetics at E13, with the results presented in Fig. 2. The heat map highlights significant differences in relative gene expression between the two groups at each specific time point, indicated by the appearance of red/brown colored bands, according to the color key. Overall, the analysis reveals a very moderate transcriptional impact of the temperature increase, particularly after 30 min, 2 h, 3 h, and 4 h of exposure, with fewer than 6 genes significantly affected out of the 81 tested. Cellular pathways related to proliferation and epigenetic modifications are among the least affected by the temperature increase, in contrast to those involved in cellular stress, thyroid hormone synthesis, or carbohydrate metabolism, for which at least 50% of the tested genes were significantly modulated. It is worth noting that the expression of many genes is never affected by the temperature increase (light pink/yellow lines, representing 44 out of the 81 tested genes, i.e., 54%). The expression of a few genes (10 out of the 81, 12.3%), such as *TET2* (epigenetics), *ATG18* and *HSP60* (cellular stress), *USP16* and *ALB* (thyroid hormone synthesis), *ACOX1* and *CEPT1* (lipid metabolism), as well as *CREBP2*, *ENO1*, and *PDHA1* (carbohydrate metabolism), was affected at two of the seven time points tested, whereas the remaining altered genes exhibited transient changes at a single time point.

**Fig. 3:**
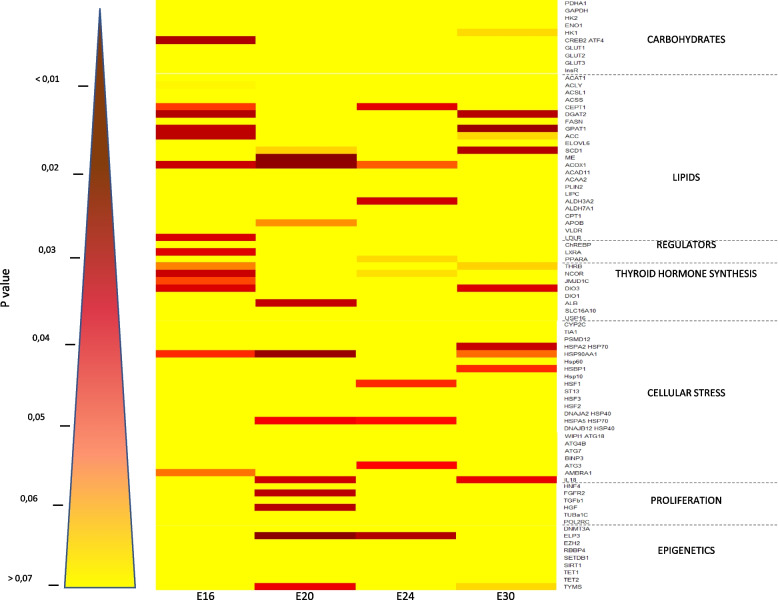
Daily kinetics of hepatic gene expression. The heatmap provides an overview of the relative expression of the 81 genes studied, presented by cell pathway for the three sampling points collected during the programming period at 3, 7, and 11 days after the onset of temperature increase (E16, E20, E24), and 3 days after the end of the programming period (E30). Student or Wilcoxon tests were applied, depending on the conditions to be met, to compare the control and TM groups for the same sampling point. The absence of a significant difference is shown in orange/yellow, while red/brown indicates a significant difference between the two groups according to the color key

The overall relative expression measured during the second trial, on daily kinetics (E16, E20, E24 and E30) is represented by a second heat map with the same color key in Fig. 3. As shown by the predominance of yellow dots, the expression of a large number of genes is not affected at all by the thermal stimulus, but 37 genes (46.6% of all potential target genes studied) are nevertheless significantly affected at least at one point. Among these target genes, 8 (9.9% of all potential target genes studied) were significantly affected at least at 2 kinetic points, with a maximum of 3 time points.

For the subsequent analysis, we grouped the 31 genes that were modulated at least once at the time points E13-1h, E16, E20, E24, and E30. To simplify the analysis, only the 1-h time point from the hourly kinetics at E13 was retained, as it is one of the time points with the highest number of temperature-responsive genes, and because the daily kinetics time points were also collected one hour after the onset of the temperature increase.

The results concerning the expression of genes involved in the energy metabolism pathway for each of these time points are shown in Table 1. The expression of 13 genes involved in carbohydrate and lipid metabolisms was significantly affected by the increase in temperature out of a total of 35 genes measured (37%). Most of them were upregulated (except for *GLUT1* at E13, and *CEPT1* at E24), suggesting an overall activation of energy metabolism. The maximum impact is measured 3 days after the start of the programming period (E16) with the increase of 8 genes, involved in each of the pathways studied. Three days after the end of the thermal stimulation (E30), at hatching, the expression of 3 genes involved in lipid synthesis (*DGAT2*, *SCD1* and *GPAT1*) was still up-regulated in programmed animals (TM) compared to control.

**Table 1:** Relative expression of temperature-modulated genes involved in liver metabolism

Pathways	Gene	Total number of genes measured	During thermal stimulus												After thermal stimulus		
			E13 (1 h)			E16 (3 days)			E20 (7 days)			E24 (11 days)			E30 (17 days)		
			Control	TM	Effect	Control	TM	Effect	Control	TM	Effect	Control	TM	Effect	Control	TM	Effect
CARBOHYDRATES																	
Transport	GLUT1	3	1.64 ± 0.85	0.82 ± 0.23	*	ns			ns			ns			ns		
Oxydation	CREB2 ATF4	7	ns			0.73 ± 0.21	1.23 ± 0.34	**	ns			ns			ns		
LIPIDS																	
Synthesis	DGAT2	10	-	-	-	0.45 ± 0.15	2.23 ± 0.62	**	ns			ns			0.83 ± 0.35	2.00 ± 0.72	**
	ACC		ns			0.62 ± 0.33	1.54 ± 0.76	*	ns			ns			ns		
	CEPT1		ns			0.72 ± 0.35	1.29 ± 0.44	*	ns			1.31 ± 0.35	0.88 ± 0.33	*	Ns		
	SCD1		-	-	-	-	-	-	ns			ns			1.10 ± 0.62	1.84 ± 0.29	**
	ME		ns			ns			0.91 ± 0.20	1.27 ± 0.19	****	ns			ns		
	GPAT1		ns			0.61 ± 0.09	1.37 ± 0.46	*	ns			ns			0.82 ± 0.25	1.53 ± 0.67	**
Oxydation	ACOX1	9	ns			0.68 ± 0.27	1.33 ± 0.53	*	0.95 ± 0.26	1.30 ± 0.29	**	0.94 ± 0.23	1.20 ± 0.47	*	ns		
	ALDH3A2		-	-	-	ns			ns			1.22 ± 0.58	2.15 ± 1.32	*	ns		
	ALDH7A1		0.86 ± 0.47	1.40 ± 0.48	*	ns			ns			ns			ns		
transport	LDLR	3	ns			0.72 ± 0.50	1.70 ± 0.84	*	ns			ns			ns		
REGULATORS																	
	LXRA	3	ns			0.78 ± 0.14	1.20 ± 0.40	*	ns			ns			ns		

The relative expression of genes involved in liver metabolism and significantly affected at least at one point by the temperature change is listed for the 5 sampling points (E13-1h, E16, E20, E24 and E30). The total number of genes measured for each cellular pathway is indicated

Student or Wilcoxon tests were applied, depending on the conditions to be met. Effect: statistical result of the comparison between the control group and the programmed group for the same time point; ns: non significant, *: *p* < 0.05, **: *p* < 0.01, ***: *p* < 0.001. « -» not determined (not enough samples to apply tests). *n* = 6 to 8

Regarding the expression of genes involved in thyroid hormone synthesis, the results shown in Table 2 highlighted an overall increase, except for *DIO3* at E30, three days after the end of thermal stimulation. E16 is still the stage at which the highest number of genes is affected by the temperature increase, with 3 genes up-regulated out of the 4 identified targets.

**Table 2:** Relative expression of temperature-modulated genes involved in thyroid hormone synthesis

Pathway	Gene	Total number of genes measured	During thermal stimulus												After thermal stimulus		
			E13 (1 h)			E16 (3 days)			E20 (7 days)			E24 (11 days)			E30 (17 days)		
			Control	TM	Effect	Control	TM	Effect	Control	TM	Effect	Control	TM	Effect	Control	TM	Effect
Thyroid hormone synthesis	NCOR	8	1.04 ± 0.26	1.29 ± 0.55	*	0.68 ± 0.22	1.33 ± 0.22	*	ns			ns			ns		
	ALB		ns			ns			1.75 ± 0.31	1.78 ± 0.31	*	ns					
	JMJD1C		ns			0.76 ± 0.28	1.31 ± 0.22	*	ns			ns			ns		
	DIO3		ns			0.68 ± 0.25	1.29 ± 0.16	*	ns			ns			2.24 ± 2.05	0.93 ± 0.14	*

The relative expression of genes involved in thyroid hormone synthesis and significantly affected at least at one point by the temperature change is listed for the 5 sampling points (E13-1h, E16, E20, E24 and E30). The total number of genes measured for this cellular pathway is indicated

Student or Wilcoxon tests were applied, depending on the conditions to be met. Effect: statistical result of the comparison between the control group and the programmed group for the same time point; ns: non significant, *: *p* < 0.05. *n* = 6 to 8

Changes in the expression of genes involved in the heat shock response, autophagy and inflammation are presented in Table 3. Among the 10 genes significantly affected at some point, half were already affected within the first hour of temperature increase, and 4 are still affected at E30, corresponding to the hatching day.

**Table 3:** Relative expression of temperature-modulated genes involved in cellular stress

Pathway	Gene	Total number of genes measured	During thermal stimulus												After thermal stimulus		
			E13 (1 h)			E16 (3 days)			E20 (7 days)			E24 (11 days)			E30 (17 days)		
			Control	TM	Effect	Control	TM	Effect	Control	TM	Effect	Control	TM	Effect	Control	TM	Effect
CELLULAR STRESS																	
Heat shock response	HSPA2- HSP70	15	2.74 ± 1.99	0.53 ± 0.18	**	ns			ns			ns			1.45 ± 0.57	0.81 ± 0.31	*
	HSP90AA1		ns			0.78 ± 0.23	1.22 ± 0.43	*	0.90 ± 0.15	1.23 ± 0.14	**	ns			1.20 ± 0.36	0.88 ± 0.20	*
	Hsp60		1.91 ± 0.69	0.71 ± 0.35	*	ns			ns			ns			ns		
	HSBP1		ns			ns			ns			ns			1.62 ± 0.73	0.87 ± 0.56	*
	HSF1		ns			ns			ns			0.92 ± 0.30	1.41 ± 0.42	*	ns		
	HSF3		1.28 ± 0.40	1.50 ± 0.63	**	ns			ns			ns			ns		
	HSPA5-HSP70		0.97 ± 0.09	1.12 ± 0.24	**	ns			0.96 ± 0.15	1.11 ± 0.23	*	0.86 ± 0.28	1.21 ± 0.33	*	ns		
Autophagy	WIPI1- ATG18	6	1.20 ± 0.26	1.28 ± 0.46	**	ns			ns			ns			ns		
	ATG3		ns			ns			Ns			0.88 ± 0.12	1.21 ± 0.41	*	ns		
Inflammation	Il18	1	ns			ns			1.84 ± 0.71	1.98 ± 1.44	*	ns			0.72 ± 0.25	2.49 ± 2.35	*

The relative expression of genes involved in cellular stress and significantly affected at least at one point by the temperature change is listed for the 5 sampling points (E13-1h, E16, E20, E24 and E30). The total number of genes measured for each cell pathway is indicated

Student or Wilcoxon tests were applied, depending on the conditions to be met. Effect: statistical result of the comparison between the control group and the programmed group for the same time point; ns: non significant, *: *p* < 0.05, **: *p* < 0.01. *n* = 6 to 8

Table 4 summarizes the results concerning the expression of genes involved in proliferation and epigenetic pathways. The 4 genes identified as targets of thermal programming in these two pathways (out of 16 tested) had their expressions modulated seven days after the onset of temperature modification (E20). Only *ELP3*, involved in the epigenetic pathway, is still affected at E24, and none of them are affected at E30 anymore.

**Table 4:** Relative expression of temperature-modulated genes involved in proliferation and epigenetic

Pathway	Gene	Total number of genes measured	During thermal stimulus												After thermal stimulus		
			E13 (1 h)			E16 (3 days)			E20 (7 days)			E24 (11 days)			E30 (17 days)		
			Control	TM	Effect	Control	TM	Effect	Control	TM	Effect	Control	TM	Effect	Control	TM	Effect
Proliferation	FGFR2	6	ns			ns			0.81 ± 0.47	1.66 ± 0.63	**	ns			ns		
	HGF		ns			ns			0.80 ± 0.36	1.51 ± 0.53	**	ns			ns		
Epigenetic	ELP3	10	ns			ns			1.55 ± 0.38	0.69 ± 0.34	***	1.03 ± 0.27	1.40 ± 0.67	**	ns		
	TYMS		ns			ns			0.81 ± 0.33	1.51 ± 0.65	*	ns			ns		

The relative expression of genes involved in proliferation or epigenetic and significantly affected at least at one point by the temperature change is listed for the 5 sampling points (E13-1h, E16, E20, E24 and E30). The total number of genes measured for each cell pathway is indicated

Student or Wilcoxon tests were applied, depending on the conditions to be met. Effect: statistical result of the comparison between the control group and the programmed group for the same time point; ns: non significant, * < 0.05, ** < 0.01, *** < 0.001. *n* = 6 to 8

### Incubation and hatching data

During the trial 2, no significant difference was observed between the two incubation treatments for embryonic mortalities (mid and late), hatchability or male proportion with average (for both groups combined) of 9.4%, 3.1%, 81.5% and 55.2% respectively (Table 5). However, ducklings subjected to thermal programing presented significantly lower body weight (48.8 against 50.2 g) and lower internal temperature (39.9 against 40.1 °C) at hatch compared to control group.

**Table 5:** Incubation and hatch measurements

	Mid-embryonic mortality (%)	Late mortality (%)	Hatchability (%)	Male proportion (%)	Weight at hatch (g)	Internal temperature at hatch (°C)
Control	10.0	3.2	78.8	55.7	50.2^a^ ± 4.5	40.1^a^ ± 0.5
TM	8.7	3.0	84.2	54.7	48.8^b^ ± 4.1	39.9^b^ ± 0.4
Number of animals in Control and TM group	501 and 554	451 and 506	433 and 475	433 and 475	237 and 255	101 and 98

Mid-embryonic mortality (E28), late mortality (E30), hatchability and male proportion were compared for the two incubation conditions by Chi^2^ tests during the second trial. Body weight was compared by t-test and internal temperature by Wilcoxon test

## Discussion

This study aimed to investigate the impact of increased incubation temperature in mule duck eggs, to gain a better understanding of short-term transcriptional and physiological changes in order to propose new tools for thermal programming in ducks. To this end, two kinetic analyses were conducted: one on an hourly scale and the other on a daily scale following the temperature increase.

### Consolidation of knowledge on thermal manipulation effects on hatching performance

At the end of the embryonic period, we measured the impact of thermal stimulation at the biological level. The increase of 1 °C between E13 and E27 for 16 h per day did not affect mid-embryonic and late mortalities, the final hatchability rate, or the sex ratio. These results partially confirm previous data showing that a discontinuous temperature increase of 1.5 °C from the 13th day of embryogenesis had no impact on late mortality and hatchability [27]. The choice of these embryonic stimulus conditions was made primarily with the objective of maintaining hatchability or sex ratio, in order to stay aligned with the programming project. Indeed, eliminating some animals at hatch (due to a decrease in hatchability or a modification of the sex ratio) would not allow to draw conclusions about the revelation of a later "programmed" phenotype, but could instead indicate a selection of the most robust animals from hatch. Once these conditions were confirmed, we then focused on the weight of the animals and their temperature at hatch. As expected, these two post-natal parameters significantly decreased with the increase in embryonic temperature, confirming numerous previously published data [14, 15, 17, 18, 28] attesting to the effective reception of the thermal stimulus and suggesting that embryonic programming indeed occurred.

### The overall analysis indicates a moderate transcriptional response to thermal manipulation

To assess the short-term transcriptomic response to this programming in the liver of embryos, a total of 11 time points were studied throughout the period of application of the thermal stimulus and three days after the end of the stimulus, at hatching. 81 potential target genes involved in different cellular pathways and selected for their potential action in the development of hepatic steatosis were analyzed using Fluidigm technology across two trials. In the first experiment, seven time points were analyzed over an hourly time course at E13, ranging from 30 min to 7 h after the onset of the temperature increase. In contrast, the second experiment focused on four time points over a daily time course, from three days after the beginning of the temperature increase (E16) to three days after the end of the programming period, at hatching (E30).

As illustrated by the overall yellow trend on both heatmaps (Figs. 2 and 3), the expression of the 81 selected genes was only moderately affected by the temperature increase. However, compiling the two kinetic studies, 46% of the pre-selected target genes (37 genes) were affected by the temperature increase at some point over the whole period, spread across all the cellular pathways studied.

The detailed analysis of five time points—1 h after the onset of the daily temperature increase during the programming period at E13, E16, E20, and E24), and three days after the end of this period (E30)—revealed different response kinetics depending on the cellular pathway studied.

### Cellular stress pathways are quickly impacted by the temperature increase

The first major group of genes to respond to the increase in temperature, within the first hour, belongs to the cellular stress pathway and primarily involves genes related to the heat stress response such as *HSPA2*-*HSP70*, *HSP60*, *HSF3* and *HSPA5*-*HSP70*. These results confirm the rapid sensitivity of this cellular pathway to different types of stress, as already shown in poultry exposed to heat shock [7, 29] or embryonic thermal conditioning [19, 23]. Given that transcriptomic changes in HSP expression can be recovered within 48 h [30, 31], it is interesting to note that the expression of several genes in this group (*HSPA2*-*HASP70*, *HSP90AA1*, *HSBP1*) was still affected by thermal programming three days after the end of the application of the thermal stimulus. This observation may reveal a different temporal response to this precise type of thermal stimulus in mule ducks, with a longer transcriptomic mark, or it may also highlight other well-described types of hatching-induced stress in birds [32–34]. However, the expression of the three genes involved in the heat shock response pathway, still significantly altered at E30, is decreased compared to the control group. This could mean that thermal programming has already increased the ducklings' ability to cope with hatching stress (particularly in terms of thermal regulation), reducing the need for cellular protection normally provided by HSPs [35, 36]. This could be reflected particularly by the expression of the *HSP90AA1* gene, a major player in the stress response system [37, 38], which is increased at E16 and E20, and decreased at E30.

Moreover, the autophagy pathway, involved in intracellular degradation under normal or stressed conditions [39, 40], also responded quickly after the increase in temperature, with an increase in *WIPI1* expression at E13 but was completely shut down at E30. Only the expression of *IL18*, a pro-inflammatory cytokine involved in the acute stress response [41], was still increased at hatching. It has been previously demonstrated that chickens subjected to embryonic thermal manipulation exhibited an early increase in *IL18* expression in the spleen during a subsequent heat shock compared to control animals [20]. This early response has been interpreted as possibly contributing to improved heat tolerance by increasing the expression of genes involved in the cell repair mechanism, and could similarly in this study, indicate an enhanced stress response during hatching. Nevertheless, the impact of embryonic thermal manipulation on the inflammatory response to hatching stress needs to be further investigated (only the expression of one gene was retained here).

### Energy metabolism is the most affected by the temperature increase

The second measurement point (E16), three days after the start of the temperature increase, revealed a marked increase in the expression of most genes involved in carbohydrate (*CREB2 ATF4*) and lipid (*DGAT2*, *ACC*, *CEPT1*, *GPAT1*, *ACOX1*) metabolisms or their regulations (*LXRA*). This overall activation of the energy pathway in the liver was accompanied by increased expression of three genes involved in thyroid hormone pathway (*NCOR*, *JMJD1C* and *DIO3*). *NCOR* (corepressor) and *DIO3* (deiodinase) are known to promote the inactive form of thyroid hormone [42–44] while *JMJD1C* is a coactivator of the thyroid hormone receptor [45, 46] and is involved in energy metabolism, in particular glycolysis and the oxidative process [47]. The concomitant increase in thyroid pathway activators and inhibitors could highlight a compensatory regulatory mechanism, but more importantly indicates that the thyroid pathway is a key target for embryonic thermal programming in mule duck. In particular, *DIO3* has already been identified as a recurrent target of embryonic thermal programming in mule ducks, both at the end of the thermal stimulus or at the end of the overfeeding period [22]. *SCD1, CEPT1* and *GPAT1* involved in lipid synthesis, or *LXRA* involved in transcriptional regulation of lipid metabolism [48] have also previously been highlighted as programming targets in the liver of thermally programmed mule ducks [16, 22, 49]. This transcriptional response, induced by the temperature change specifically during the embryonic period corresponding to the peak maturation of liver functions [33], may reflect an adaptation to the metabolic demands observed at this stage and represent an initial mechanism of programming. Besides, although the peak of the transcriptional response to the thermal stimulus is clearly visible three days after the onset of the temperature increase (E16), the expression of several genes is affected throughout embryogenesis and even after hatching (E30). This sustained transcriptional response demonstrates that hepatic metabolism is directly affected by thermal stimulus during embryogenesis, and confirms that metabolic programming is measurable.

### Cell proliferation and epigenetic marks pathways are moderately and transiently activated by thermal manipulation

Finally, the last two groups of genes to be affected by the rise in incubation temperature, predominantly at E20, are those involved in cell proliferation and epigenetics. *FGF* and *HGF* are two growth factors that play fundamental role in liver development [50, 51] or liver regeneration [52–55]. Previously, high expression of *HGF* and *FGF* receptor 2 (*FGFR2*) was measured between days 12 and 20 of embryogenesis in mule ducks [33], highlighting their possible activity in the development and growth of liver tissue at this stage. It was suggested that a change in temperature during this period might influence the final number of liver cells in the mature organ, as previously shown in chicken muscle cells [11, 18]. This hypothesis, supported here by a temperature-induced increase in *FGFR2* and *HGF* gene expression, should now be verified by measurements of liver size or cell number. Indeed, although hyperplasia does not appear to contribute to fatty liver enlargement during overfeeding [56], it is conceivable that an increased number of hepatocytes at hatch may promote liver fattening, and then participate to the programming phenotype observed in overfed mule ducks [16, 17].

Then, only 2 out of the 9 tested genes involved in epigenetics mechanisms are affected by the thermal manipulation at E20 and E24. First, *TYMS* is an actor in one-carbon metabolism that provides methyl groups known to be linked to epigenetic mechanisms [57, 58]. The increase in its expression several days after the start of the incubation temperature change could reflect a transient activation of the DNA methylation process that could be involved in the programmed phenotype measured at hatching, but also when ducks are overfed. Secondly, elongator protein 3 (*ELP3*) is a histone acetyltransferase which facilitates transcript elongation by opening access for the RNA polymerase II to numerous genes [59, 60]. In humans, *ELP3* has been shown to regulate *HSP70* gene expression, involved in heat stress response [61], notably through modulation of histone H3 acetylation [62]. Here in duck embryos, we first measured a decrease in *ELP3* mRNA expression 7 days after the onset of temperature increase, followed by an increase after 11 days, confirming the sensitivity of this histone acetyltransferase to temperature change. However, these changes in *ELP3* gene expression were not accompanied by a similar modulation of *HSP70s* mRNA, suggesting that in ducks, *ELP3* and *HSP70* are not directly linked. To better understand the role of *ELP3* during temperature increases in ducks, it would now be interesting to measure histone acetylation activity under these conditions, as well as later in the animal's life to assess the persistence of these potential epigenetic marks and their actual role in the programmed phenotype observed.

## Conclusion

Although sporadically, all the metabolic pathways suspected of being targets of embryonic thermal programming were affected by the thermal stimulus at different time points during the temperature increase and even up to 3 days afterwards. This study represents only one aspect of the mechanisms involved in metabolic programming induced by an increase in incubation temperature in ducks. Further studies will be required to determine the modulations of protein expression corresponding to the identified mRNAs, enzyme activities, or epigenetic marks. Moreover, the targeted analysis of 'candidate' genes using the Fluidigm technique provides only a partial answer to the question of which metabolic pathways are directly affected by temperature. A more comprehensive approach, such as RNA sequencing, should therefore be considered for future investigations.

However, this study has highlighted for the first time a short-term transcriptional response kinetics of hepatic cells to embryonic thermal stimulus. In particular, it revealed the rapid and sustained induction of heat shock proteins, the activation of a large number of genes involved in metabolism, and the low proportion of genes involved in epigenetic processes affected by temperature. These results could serve as new tools to better understand the mechanisms of embryonic thermal programming.

## Supplementary Information

Supplementary Material 1

Supplementary Material 2

## Data Availability

The datasets used and/or analysed during the current study are available in the GEO database under the reference GSE303902 (publicly accessible from August 3, 2025) [https://www.ncbi.nlm.nih.gov/geo/query/acc.cgi?acc = GSE303902](https:/www.ncbi.nlm.nih.gov/geo/query/acc.cgi?acc = GSE303902).
