# Supplementary material for: Short-term impacts of embryonic thermal manipulation in mule duck, a kinetic study: new tools for metabolic programming
Source: BMC Genomics. 2025 Nov 7;26:1010. doi: 10.1186/s12864-025-12192-7 (PMC12595638; doi:10.1186/s12864-025-12192-7)
Supplement: Supplementary file 1 — Supplementary Material 1 [file 12864_2025_12192_MOESM1_ESM.docx]

**Supplement table 1. Primers used in the Fluidigm described in the previous results**

The primers that revealed an impact of the temperature change on the expression of the corresponding gene are listed here (31 primers).
