# Supplementary figures and images for: Short-term impacts of embryonic thermal manipulation in mule duck, a kinetic study: new tools for metabolic programming
Source: BMC Genomics. 2025 Nov 7;26:1010. doi: 10.1186/s12864-025-12192-7 (PMC12595638; doi:10.1186/s12864-025-12192-7)

**Supplemental table 2 :**


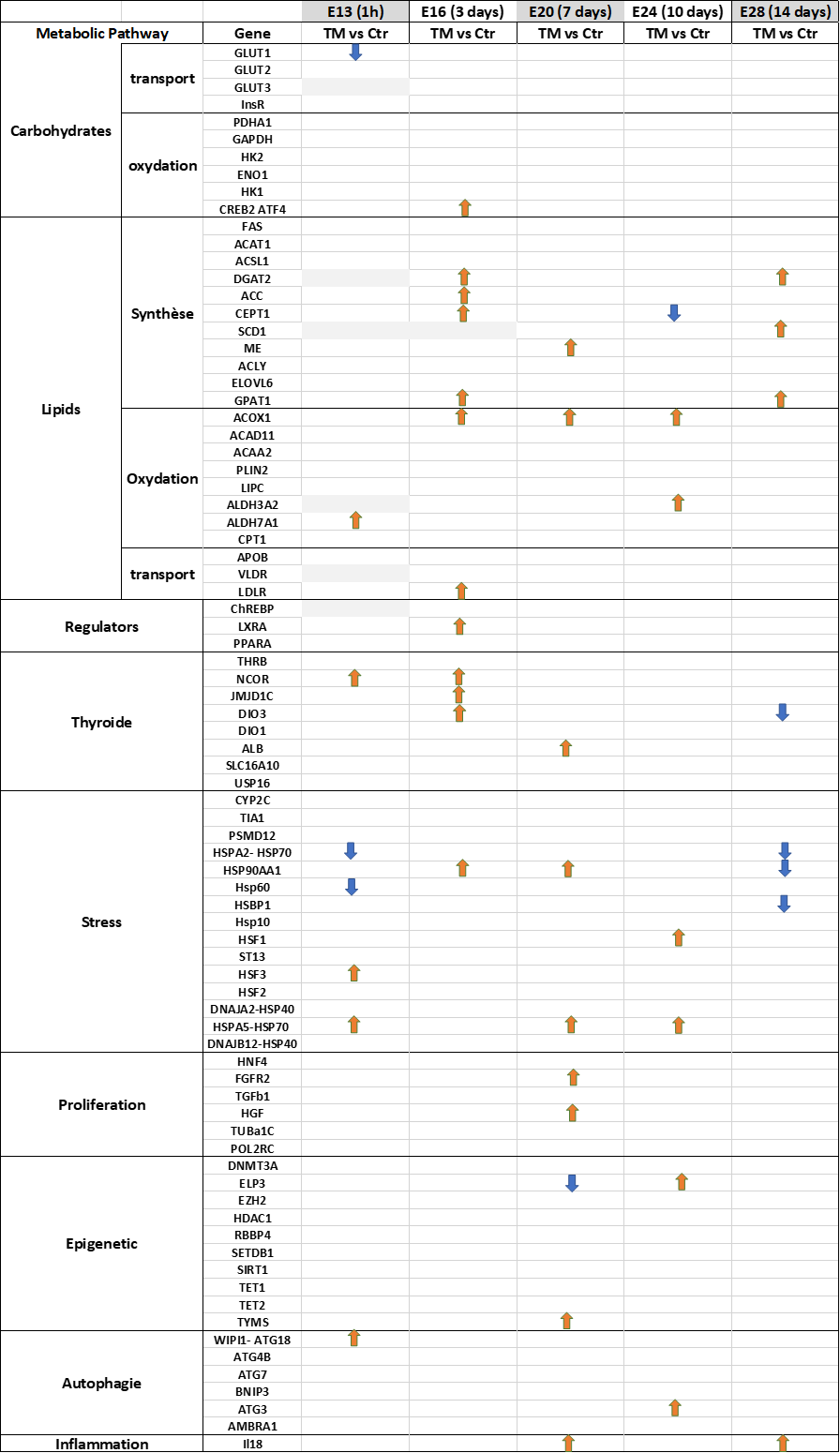

Supplement: Supplementary file 2 — Supplementary Material 2 [file 12864_2025_12192_MOESM2_ESM.docx]
